# In *Arabidopsis thaliana*, RNA-Induced Silencing Complex-Loading of MicroRNAs Plays a Minor Regulatory Role During Photomorphogenesis Except for miR163

**DOI:** 10.3389/fpls.2022.854869

**Published:** 2022-07-13

**Authors:** Lóránt Lakatos, Gergely Groma, Daniel Silhavy, Ferenc Nagy

**Affiliations:** ^1^Laboratory of Photo and Chronobiology, Biological Research Centre, Institute of Plant Biology, Eötvös Loránd Research Network, Szeged, Hungary; ^2^Dermatological Research Group, University of Szeged, Szeged, Hungary

**Keywords:** *Arabidopsis thaliana*, photomorphogenesis, reprogramming of gene expression, miRNA, efficiency of AGO loading, miRNAome, RISC-loaded miRNAome

## Abstract

The shift of dark-grown seedlings to the light leads to substantial reprogramming of gene expression, which results in dramatic developmental changes (referred to as de-etiolation or photomorphogenesis). MicroRNAs (miRNAs) regulate most steps of plant development, thus miRNAs might play important role in transcriptional reprogramming during de-etiolation. Indeed, miRNA biogenesis mutants show aberrant de-etiolation. Previous works showed that the total miRNA expression pattern (total miRNAome) is only moderately altered during photomorphogenesis. However, a recent study has shown that plant miRNAs are present in two pools, biologically active miRNAs loaded to RISC (RNA-induced silencing complex-loaded) form while inactive miRNAs accumulate in duplex form upon organ formation. To test if RISC-loading efficiency is changed during photomorphogenesis. we compared the total miRNAome and the RISC-loaded miRNAome of dark-grown and de-etiolated *Arabidopsis thaliana* seedlings. miRNA sequencing has revealed that although regulated RISC-loading is involved in the control of active miRNAome formation during de-etiolation, this effect is moderate. The total miRNAomes and the RISC-loaded miRNAomes of dark-grown and de-etiolated plants are similar indicating that most miRNAs are loaded onto RISC with similar efficiency in dark and light. Few miRNAs were loaded onto RISC with different efficiency and one miRNA, miR163, was RISC-loaded much more effectively in light than in dark. Thus, our results suggest that although RISC-loading contributes significantly to the control of the formation of organ-specific active miRNA pools, it plays a limited role in the regulation of active miRNA pool formation during de-etiolation. Regulated RISC-loading strongly modifies the expression of miRNA163, could play a role in the fine-tuning of a few other miRNAs, and do not modify the expression of most miRNAs.

## Introduction

Light regulates many steps of plant development including germination, seedling development, shade avoidance, and flowering (Deng and Quail, 1999; Casal, 2012; Łabuz et al., 2012). The shift of dark-grown seedlings into light leads to substantial reprogramming of gene expression manifests in dramatic developmental changes. The molecular mechanisms of light-controlled seedling development (photomorphogenesis) are well studied. Light-sensitive photoreceptor molecules sense the intensity and quality of the light and trigger different signaling pathways leading to rapid transcriptional reprogramming and changes in protein stabilities (Legris et al., 2019; Cheng et al., 2021). The function of regulatory RNAs in the control of photomorphogenesis is less understood. MicroRNAs (miRNAs) are single-stranded, ∼21–24 nt long non-coding RNAs that play a critical role in post-transcriptional gene regulation in plants (Naqvi et al., 2012; Wang et al., 2019). miRNAs are produced from long, stem-loop structured RNA Polymerase II transcripts called primary miRNAs (pri-miRNAs) by the microprocessor complex (Chen, 2005; Yu et al., 2017). The core components of the microprocessor are the DICER-LIKE1 (DCL1) RNase III enzyme, the zinc finger protein SERRATE (SE), and the double-stranded RNA binding protein HYPONASTIC LEAVES 1 (HYL1) (Kurihara and Watanabe, 2004; Kurihara et al., 2006; Yang et al., 2006). DCL1, the key factor of the microprocessor, first produces precursor-miRNAs (pre-miRNAs) from the pri-miRNAs and then cleaves the pre-miRNAs at sites determined by structural features, thereby producing miRNA-miRNA* duplexes (Iwata et al., 2013). The duplexes are methylated at their 3′ termini by the HUA ENHANCER1 (HEN1) methyltransferase (Yu et al., 2005). The methylated miRNAs are loaded in a single-stranded form onto the ARGONAUTE 1 (AGO1) protein (or less frequently onto one of the many other AGO proteins) (Carbonell and Carrington, 2015) and form presumably with other proteins the functional RNA-Induced Silencing Complex (RISC) (Azevedo et al., 2010; Giner et al., 2010; Dalmadi et al., 2019). The miRNAs can be transported into the cytoplasm in the miRNA-AGO complex or the miRNA-miRNA* duplex (Yu et al., 2017; Bologna et al., 2018; Wang et al., 2019). In the cytoplasm, miRNA guides AGO1 to the mRNAs that show sequence complementarity with the small RNA to silence the target transcript via mRNA cleavage or translational repression. miRNA expression is strictly regulated, transcription of miRNA genes, processing of pri-miRNAs, selection of AGO protein, and stability of miRNAs are precisely controlled (Xie et al., 2010; Naqvi et al., 2012; Li and Yu, 2021). Moreover, a recent study has shown that in *Arabidopsis thaliana* production of a biologically active miRNA pool can be controlled at the AGO-loading step (Dalmadi et al., 2019). Small RNA-sequencing from the crude extract and gel-filtrated fractions of the crude extract revealed that miRNAs accumulate in two major pools, one that co-fractionates with AGO1 at the high molecular (mol.) weight fractions and a second pool that is present at the low mol. weight fractions. It is proposed that the miRNAs, which co-fractionate with AGO1 are present in the potentially active RISC-loaded form (but see section “Discussion”), while the miRNAs that accumulate at the low mol. weight fractions are present in inactive, miRNA duplex form (Dalmadi et al., 2019). Certain miRNAs mainly accumulate in the active RISC-loaded form, while other miRNAs are predominantly present as duplexes in low mol. weight fractions. The difference between the total miRNA pool (miRNAome) and the RISC-loaded miRNA pool (RISC-loaded miRNAome) is defined as RISC-loading efficiency. A comparison of RISC-loaded fractions of *Arabidopsis* leaves and flowers demonstrated that the RISC-loading efficiency of many miRNAs is organ specifically regulated (Dalmadi et al., 2019).

MicroRNAs regulate many steps of plant development, thus it is expected that miRNAs are also involved in the control of photomorphogenesis. Indeed, *Arabidopsis* mutants affected in miRNA expression show aberrant photomorphogenesis responses. Photomorphogenesis is altered in certain miRNA mutants, for example, miR167b and miR848 act as negative, whereas miR319b and miR160b as positive regulators of photomorphogenesis (Sun et al., 2018). Moreover, the *dcl1-9* and *hyl1-2* miRNA biogenesis mutants were hypersensitive to red light in the hypocotyl elongation assay (Sun et al., 2018). Furthermore, if dark-grown *Arabidopsis* seedlings are de-etiolated (shifted to light), the DCL1, HYL1, and SE microprocessor proteins accumulate to high levels and the pri-miRNA levels of most miRNAs are enhanced (Choi et al., 2020; Bhagat et al., 2022; Jung et al., 2022). The expression of microprocessor components is under complex regulation, for instance, altered transcription, phosphorylation, and protein stability all contribute to the increased HYL1 level during de-etiolation (Choi et al., 2020; Sacnun et al., 2020; Bhagat et al., 2022; Jung et al., 2022). However, high throughput miRNA sequencing (miRNA-seq) revealed that although pri-miRNA levels and microprocessor concentrations are increased during photomorphogenesis, the total miRNAome is only moderately altered (Choi et al., 2020). The total miRNA level remains stable during photomorphogenesis because light stabilizes the FORKHEAD-ASSOCIATED DOMAIN 2 (FHA2) microprocessor suppressor protein, thus miRNA processing is slow despite the enhanced microprocessor concentration (Park et al., 2021). The miRNA composition has also changed moderately during photomorphogenesis. Although many miRNAs accumulate significantly different in the dark-grown and de-etiolated plants, the changes are modest and not consistent between studies (Shikata et al., 2014; Chung et al., 2016; Choi et al., 2020; Li and Yu, 2021). The exception is miR163, which is a light-inducible miRNA that accumulates dramatically during photomorphogenesis (Chung et al., 2016; Choi et al., 2020; Li and Yu, 2021). Upregulation of miR163 is physiologically relevant, and the miR163-mediated silencing of 1,7-PARAXANTHINE METHYLTRANSFERASE (PMXT1) is required for normal primary root elongation during photomorphogenesis (Chung et al., 2016; Li and Yu, 2021).

It is controversial that miRNA biogenesis factors are important for normal photomorphogenesis (Cho et al., 2014; Sun et al., 2018), while total miRNAome is only moderately altered during de-etiolation (Choi et al., 2020). It was also reported that although in de-etiolated plants the total miRNAome changed only slightly, miRNA activity might be enhanced. During de-etiolation, the expression of certain miRNA target transcripts was declined, while their 3′ cleavage products accumulated (Lin et al., 2017). If the RISC-loading is more efficient in de-etiolated than in dark-grown plants, the biologically active, RISC-loaded miRNAomes would be more different than the total miRNAomes and could explain how light can enhance the target cleavage activity of miRNAs. To test this assumption, we wanted to comparatively study the changes of total miRNAome and RISC-loaded miRNAome during photomorphogenesis. Crude extracts were prepared from dark-grown and de-etiolated *Arabidopsis* seedlings and these extracts were gel-filtrated to separate the RISC-loaded and non-loaded miRNAs. Then miRNA-seq assays were conducted from the crude extracts and from the AGO containing gel-filtrated fractions to analyze the changes of total miRNAomes and the RISC-loaded miRNAomes during photomorphogenesis. We found that both total miRNAome and RISC-loaded miRNAome differed moderately between dark-grown and de-etiolated seedlings. Our results suggest that most miRNAs are loaded with similar efficiency in both dark-grown and de-etiolated samples, few miRNAs are selectively loaded but the differences are modest. The exception was miR163 was much more efficiently loaded onto RISC in de-etiolated plants than in dark-grown seedlings. These data suggest that during photomorphogenesis, regulated RISC-loading plays a critical role in the formation of an active miR163 pool and contributes to the control of the biologically active pool of a few other miRNAs. Thus, we conclude that selective RISC-loading does not substantially control the formation of active miRNAome (except for miR163) during de-etiolation, and instead, it could play a role in the fine-tuning of miRNA expression.

## Materials and Methods

### Plant Material and Growth Conditions

*Arabidopsis thaliana* Col-0 seeds were put on plates containing 1 × Murashige-Skoog medium supplemented with 1 w/v sucrose and kept in the dark at 4°C for 3 days, and illuminated with white light for 4 h. Then plates were kept in dark at 22°C. On day 5, half of the plates were transferred to white light for 72 h (de-etiolation), while the rest of the plates were kept in dark for 72 h (referred to as light and dark samples, respectively). Finally, 8 days old seedlings were collected and frozen in liquid nitrogen.

### Extracts Preparation and Gel-Filtration

Crude extracts used for gel-filtration (and for immunoprecipitation, see later) were prepared in a buffer containing 50 mM Tris pH 7.0, 10 mM NaCl, 5 mM MgCl_2_, and 4 mM DTT from dark and light samples as described (Dalmadi et al., 2019). Part of the crude extract was stored and used as input. The 400-μl crude extracts were loaded onto a Superdex HR-200 10/30 GL column (Merck Inc., United States). The void volume of the column (7 ml) was released, and then 36 fractions (500 μl) were collected. Even-numbered fractions were used to isolate RNA and odd-numbered fractions were used for protein isolation.

### RNA Isolation, Small RNA Northern Hybridization

Inputs and gel-filtrated fractions were extracted with phenol-chloroform-isoamyl alcohol (500:125:1, pH 4.6) and then 1/10th volume of 3M NaOAc was added. RNA was precipitated with 1 vol. of isopropyl alcohol. Resuspended RNAs were used for miRNA-seq and for Northern blot assays. RNA was separated in 8M urea 12% acrylamide (38% acrylamide, 2% bis-acrylamide) 1 × TBE gels and transferred to Hybond + membrane (GE Healthcare, United States). Locked nucleic acid (LNA) (Merck Inc., United States) containing oligonucleotides labeled at the 5′ end with biotin corresponding to the reverse complement of miR163, miR159b, and miR168a were used for miRNA detection. Hybridization and detection were carried out with the North2South™ Chemiluminescent Hybridization and Detection Kit (Thermo Fisher Scientific 17097) according to the instructions of the manufacturer.

### Protein Isolation and Western Blotting

Fractions of the gel-filtration experiment were precipitated with four volumes of ethanol, centrifuged with 14,000 × *g* at 4°C for 10 min, and resuspended in 20 μl of 2 × Laemmli buffer. Resuspended fractions and input samples (30 μl of crude extract) were loaded into 10% acrylamide SDS gels, after separation proteins were transferred to PVDF membranes (Bio-Rad, CA, United States; Immun-Blot PVDF Membrane, 1620177). Incubations were carried out with the AGO1 (Agrisera, Sweden AS09 527), actin (Agrisera, Sweden AS13 2640), and histone H3 (Abcam, United States ab1791) antibodies.

### Immunoprecipitations

Crude extracts were prepared identically for immunoprecipitation (IP) and gel-filtration. IPs were carried out with the AGO1 antibody (Agrisera Sweden AS09 527) as described (Dalmadi et al., 2019). Samples were taken from inputs (In), supernatant (SN), and the third wash off the resin (W#3). Half of the eluates were diluted in 100 μl IP2 buffer (50 mM TRIS pH 7.5, 150 mM NaCl, 5 mM MgCl2, 4 mM DTT) and then RNA was isolated as described earlier. The second half of the eluate was denatured in 2 × Laemmli buffer. Northern and Western blotting were used to detect miRNAs and proteins as described earlier.

### Cell Separation

The separation of nuclei from cytoplasm was performed as described (Dalmadi et al., 2019). One gram of seedling was extracted in 10 ml extraction buffer (10 mM Tris pH 7.5, 1.14 M sucrose, 5 mM MgCl2, 7 mM mercaptoethanol), then the crude extracts were filtered through three layers of Miracloth. After 10 min centrifugation at 4°C at 900 × *g*, samples were taken from the supernatant and hereafter referred to as cytoplasmic extracts. Pellet was washed three times with extraction buffer supplemented with 0.15% Triton X-100 and centrifuged with the same parameters. ^100 μl of the supernatants and 200 μl of the third wash were used for RNA and protein extractions. The pelleted washed nuclei were resuspended in a 5 ml solution of lysis buffer (50 mM Tris pH 7.5, 5 mM MgCl_2_, and 5 mM KCl), and then 50–50 μl were used for RNA and protein extraction.

### High Throughput MicroRNA Sequencing

To characterize total miRNAomes and RISC-loaded miRNAomes from dark-grown and de-etiolated (dark and light, respectively) *Arabidopsis* seedlings, high throughput miRNA sequencing (miRNA-seq) was conducted from crude input extracts of dark and light samples (referred to as D Input and L Input) and from AGO1 containing high mol. weight gel-filtrated fractions of D and L extracts (referred to as D RISC and L RISC for Dark RISC-loaded and Light RISC-loaded samples). AGO1 containing fractions (fraction 3–6) were combined. For each condition, four biological replicas were sequenced. Small RNA libraries have been prepared with QIAseq miRNA Library Kit for Illumina NGS systems according to the manufacturer’s protocol (Qiagen, Germany). Sequencing has been performed on Illumina NextSeq 500 platform with 1 × 75 bp SE reads.

Raw sequencing data have been processed first with FastQC to assess the overall quality of sequences, then Unique Molecular Identifiers have been extracted by using UMI_tools (v1.1.1) (Smith et al., 2017). Reads have been filtered using Biopython to include reads that are min. 18 bps and max. 30 bps long. Alignment of reads was performed to NCBI GCA_000001735.1_TAIR10 using Bowtie (v1.2.2) (Langmead et al., 2009). The resulting SAM file has been converted to BAM using the SAMtools view option (v1.10) (Danecek et al., 2021). The BAM file has been deduplicated by grouping reads with the exactly same UMIs together with UMI_tools (v1.1.1). Features have been counted and annotated according to miRbase v22 by feature counts (Liao et al., 2014) (DESeq2 has been used to normalize samples; Love et al., 2014).

### Bioinformatical Analysis

The four miRNA sequencing datasets (D Input, L input, D RISC, and L RISC) were uniformly processed. A total of 101 miRNAs were identified (Supplementary Table 1), and then miRNAs that were below 10 copies in at least 50% of the four different conditions were excluded from the dataset and were not subjected for comparison. miRNA*s (*refers to the unloaded miRNA strand) were also excluded, thus 34 miRNAs were included in the analysis. The data were normalized to the corresponding total amount of all miRNAs identified in the given experiment. Relative total amounts and RISC-loaded amounts were calculated for all miRNAs in light and dark and were compared. The student’s *t*-test was also used to test for differential expression between light and dark conditions of total miRNA amounts as well as for RISC-loaded miRNA amounts. *P*-values lower than 0.05 were considered statistically significant.

## Results

Previous results have shown that although miRNA biogenesis mutants show aberrant photomorphogenesis (Cho et al., 2014; Sun et al., 2018), the total miRNAome is only moderately changed (Choi et al., 2020). Here, we wanted to comparatively study the changes of total miRNAome and RISC-loaded miRNAome during photomorphogenesis.

### Gel-Filtration Efficiently Separates the RNA-Induced Silencing Complex-Loaded and Unloaded MicroRNAs of the Seedling Extracts

**FIGURE 1:**
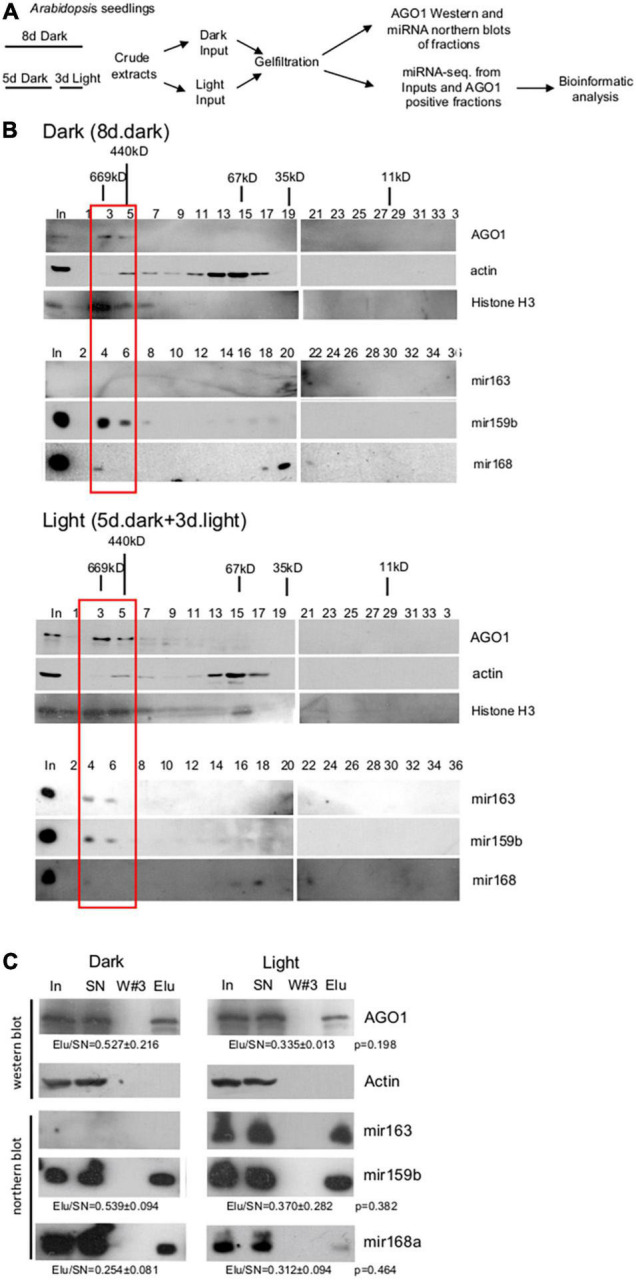
Size-selective separation of miRNAs. **(A)** Schematic representation of the experimental plan. **(B)** Gel-filtration efficiently separates the RISC-loaded and unloaded miRNAs. Crude extracts were prepared from *Arabidopsis* seedlings grown for 8 days at dark (Dark) and from de-etiolated seedlings (Light), which were grown for 5 days at dark and then for 3 days in white light. Gel-filtrated fractions were collected and Western blot assays were conducted with AGO1, histone H3, and actin antibodies or RNAs were isolated from the fractions, and then Northern blot experiments were carried out with biotin-labeled miR163, miR159b, and miR168 LNA oligos. Black sticks represent protein size markers in kilodalton (kDa). The first lanes represent the protein and RNA inputs isolated from the crude extracts (In). The red bracketed fractions (3–6) were combined and used for miRNA-sec. Note that AGO1, miR163, and miR159b accumulate in high mol. Weight fractions, while miR168 is mainly present in low mol. Weight fractions and that miR163 is not detectable in the dark samples. **(C)** miR163, miR159b, and miR168 are associated with AGO1. Crude extracts of dark and light samples were used for immunoprecipitation (IP) using the AGO1 antibody. RNA samples isolated from the input (In), the supernatant (SN), the third wash (W#3), and the pellet (Elu) of the AGO1 IP were used for Northern blot assays. Proteins were isolated from the same sources and subjected to Western blotting to detect AGO1 and the cytoplasmic control actin protein. To estimate the efficiency of AGO1 IP and the ratio of AGO1-loaded fraction of miR168 and miR159, the ratios of Elu and SN signals (Elu/SN) of the light and dark samples were calculated for AGO1 and for the two miRNAs (from three replicas), and then the light and dark Elu/SN values were compared. Elu/Sn mean values and ± standard deviations are shown. The Elu/SN values were not significantly different between dark and light samples (see *p*-values). Note that AGO1 IP was partial, thus it is a semi-quantitative assay.

To compare total miRNAomes and RISC-loaded miRNAomes between dark-grown and de-etiolated plants, we should efficiently separate RISC-loaded and unloaded miRNAs. To establish an effective separation system, crude extracts were prepared from *Arabidopsis* seedlings grown for 8 days in the dark or shifted on day 5 from dark to white light for 3 days (referred to as dark and light samples, respectively), and then a part of dark and light samples was subjected to gel-filtration (Figure 1A). Fractions were collected and subjected to AGO1, histone H3, actin western blot, and miR163, miR159b, and miR168a small RNA gel blot assays (Figure 1B). The studied proteins and miRNAs accumulated similarly in the dark and light samples except that miR163 was detectable only in the light sample. This confirms that miR163 is a light-inducible miRNA. The crude extracts contain both nuclear and cytoplasmic fractions and the nuclear control H3 accumulated in the high mol. weight fractions, while the cytoplasmic control actin was present in the medium mol. weight fractions (Figure 1B). As expected, AGO1 was also present in the high mol. weight fractions. miR159b and miR163 RNAs were co-fractionated with AGO1 in the high mol. weight fractions. By contrast, miR168 accumulated to low levels in the AGO1-containing fractions, and it was mainly present in the low mol. weight fractions (Figure 1B). These low mol. weight fractions likely correspond to the cytoplasmic free miRNA duplexes (Dalmadi et al., 2019).

To confirm that the miRNAs that co-fractionated with AGO1 could be complex with AGO1, co-immunoprecipitation studies were conducted. AGO1 IP was similarly efficient from dark and light samples. However, a significant amount of AGO1 remained in the supernatant showing that AGO1 IP was partial. As we expected, all three miRNAs can be immunoprecipitated with the AGO1 antibody (Figure 1C). MiR159 and miR168 were AGO1 immunoprecipitated with comparable efficiency in dark and light samples (miR163 is not present in the dark samples) suggesting that these two miRNAs are similarly represented in the immunoprecipitated fraction of AGO1 in the dark and light samples.

Taken together, our gel-filtration results indicate that miR159 and miR163 were efficiently loaded onto AGO1 (or less likely to other AGOs that also accumulated in the high mol. weight fractions), while miR168 was inefficiently loaded onto AGOs, and it was mainly present as miRNA duplex. In mature *Arabidopsis* leaves and flowers, AGO1 was also present in the high mol. weight fractions, miR159 and miR163 were efficiently loaded onto AGO1, and miR168 was predominantly present in miRNA duplex form (Dalmadi et al., 2019). Thus, the studied miRNAs show similar AGO loading patterns in the dark-grown and de-etiolated seedlings as well as in the different organs of full-grown plants.

### Total MicroRNA Levels Are Moderately Changed During Photomorphogenesis Except for miRNA163

**FIGURE 2:**
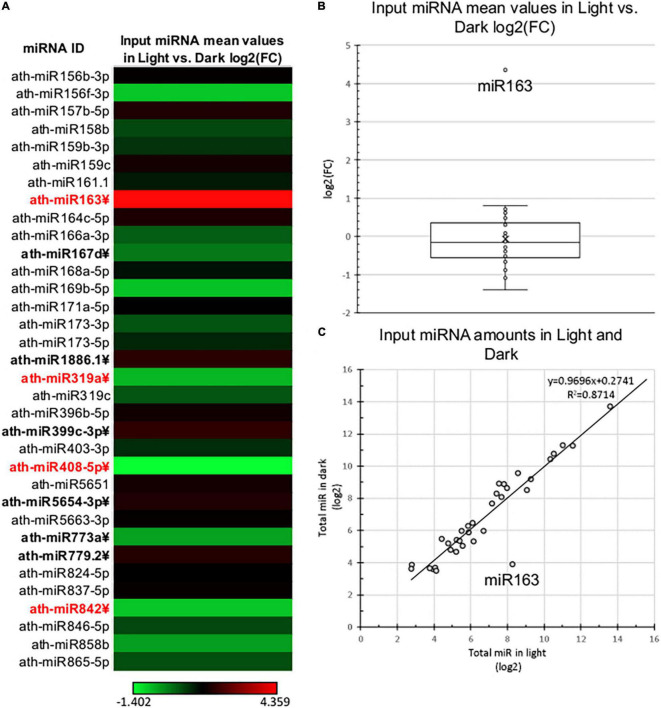
Total miRNA expression of dark-grown and de-etiolated *Arabidopsis* seedlings. **(A)** The heat map of relative expressional differences of miRNAs of dark-grown (Dark) and de-etiolated (Light) seedlings [log2(FC)]. miRNAs that express significantly different are in bold letters, which show at least ±2-fold difference are highlighted in red. **(B,C)** Relative expressional distribution of identified miRNAs in light and dark samples. The salient data point represents miR163 in both **(B,C)**.

To comparatively study the changes of total and RISC-loaded miRNAomes during photomorphogenesis (Figure 1A), miRNA-seq was conducted from crude input extracts of dark and light samples and the AGO1 containing gel-filtrated fractions of dark and light extracts (referred to as D Input, L Input, D RISC-loaded, and L RISC-loaded samples, respectively). Four biological replicas were studied. Principal component analyses show that the samples are relevantly different (Supplementary Figure 2), one component separated the dark and light samples, while the second component separated the input and RISC-loaded samples. The identical samples were grouped closely together. After excluding the miRNA* strands and the miRNAs that were very poorly expressed (see section “Materials and Methods”), we analyzed the expression of 34 miRNAs. First, we compared the miRNAome of D and L inputs. In line with previous studies (Choi et al., 2020; Li et al., 2021), we found that the miRNAomes of D and L inputs were moderately different and 10/34 miRNAs accumulated significantly different (Figure 2 and Supplementary Table 2A). However, the changes are modest, only four miRNAs (miR163, miR319a, miR408, and miR482) showed more than 2-fold differences. Three of them, miR319a, miR408, and miR482, were underrepresented in the L input (0.49, 0.38, and 0.47-fold). miRNA163 was exceptional, it accumulated to low levels in the D input, while it was dramatically increased (∼20.5-fold) in the L input (Supplementary Table 2A). These results are consistent with previous results showing that during photomorphogenesis, miRNA163 was dramatically upregulated, while the expression of other miRNAs was changed slightly (Choi et al., 2020; Li et al., 2021).

### During Photomorphogenesis, the AGO-Loading Efficiency of miR163 Is Strongly Enhanced While the Loading Efficiency of Other MicroRNAs Is Only Slightly Changed

**FIGURE 3:**
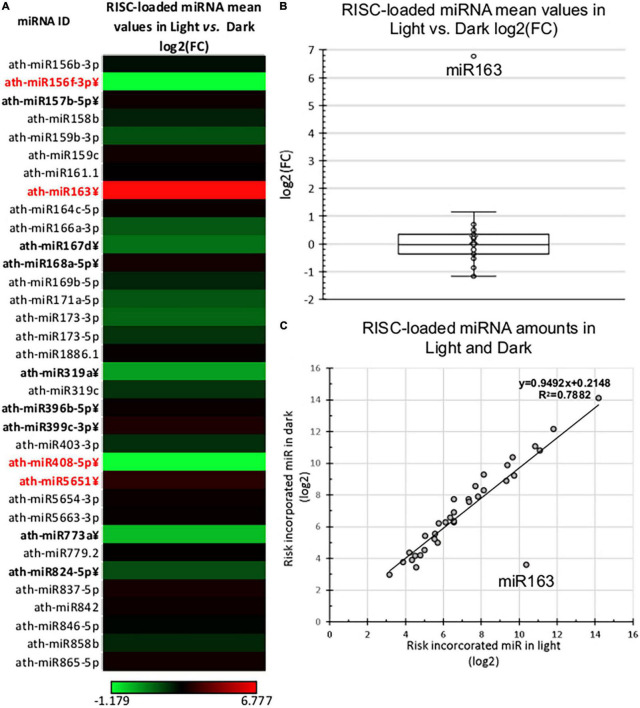
RISC-loaded miRNA expression of dark-grown and de-etiolated *Arabidopsis* seedlings. **(A)** The heat map of the relative expressional ratio of identified RISC-loaded miRNAs in dark-grown (Dark) and de-etiolated (Light) samples [log2(FC)]. miRNAs that express significantly different are in bold letters, which show at least ±2-fold difference are highlighted in red. **(B,C)** Relative RISC-loaded distribution of identified miRNAs in light and dark samples. The salient data point represents miR163 in both **(B,C)**.

Next, we compared the RISC-loaded miRNAomes of dark and light samples. The RISC-loaded miRNAomes were also moderately different, 12/34 miRNAs accumulated significantly different and 6-6 miRNAs were overrepresented in the RISC-loaded light and dark samples (Figure 3 and Supplementary Table 2B). However, only four miRNAs show more than 2-fold differences, miR408 and miR156f-3p were less abundant (0.44 and 0.44-fold), while miR565 (2.2-fold) and miR163 were more abundant in the light RISC-loaded samples. Notably, miR163 was dramatically overrepresented (109.66-fold) in the RISC-loaded de-etiolated sample relative to the dark-grown RISC-loaded sample (Figure 3 and Supplementary Table 2B).

**FIGURE 4:**
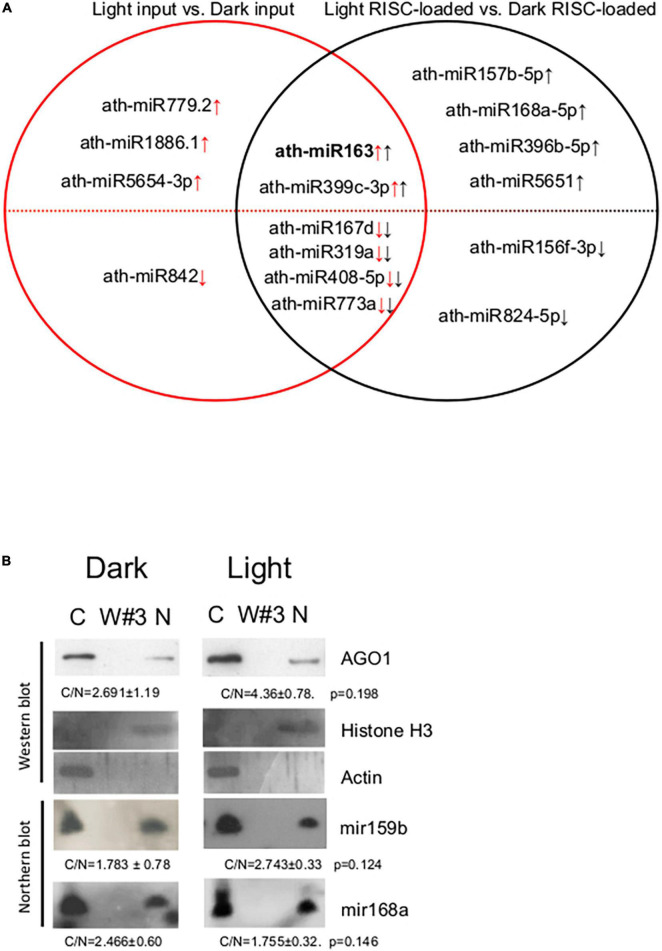
Distribution of AGO1 and miRNAs in the crude extracts of *A. thaliana* seedlings. **(A)** Set diagram of miRNAs that differ between light input and dark input samples, and between light RISC-loaded and dark RISC-loaded samples. Arrows indicate the direction of change in light vs. dark comparison in inputs (red) or RISC-loaded samples (black). **(B)** Nuclear and cytoplasmic fractions were separated from crude extracts of dark-grown (Dark) and de-etiolated (Light) seedlings. RNA and protein samples were isolated from the cytoplasmic fraction (C), the third wash (W#3), and the isolated nuclei (N) and then subjected to Western and Northern analysis. AGO1, nuclear control histone H3, and cytoplasmic control actin proteins were detected. miR168 and miR159b were detected by Northern hybridization. To quantify the distribution, the ratios of cytoplasmic and nuclear signals (C/N) of the light and dark samples were calculated and compared (from three replicas). C/N mean values and ± standard deviations are shown. The C/N values were not significantly different between dark and light samples (see *p*-values).

To assess the role of RISC-loading efficiency in the formation of biologically active, RISC-loaded miRNAome during photomorphogenesis, we compared the total miRNAomes and the RISC-loaded miRNAomes. The 10 miRNAs were accumulated differently between light and dark inputs, while 12 miRNAs were between RISC-loaded light and dark samples (Figure 4A and Supplementary Tables 2A,B). We did not find any miRNA that showed significant but opposite accumulation in the inputs and the RISC-loaded samples. Four miRNAs (miR842, miR5654-3p, miR1886.1, and miR779.2) were present in significantly different amounts in the inputs but not in the RISC-loaded samples, while six miRNAs (miR824-5p, miR156f-3p, miR5651, miR396b-5p, miR168a-5p, and miR157b-5p) accumulated differently in the RISC-loaded samples but not in the inputs (Figure 4A, Supplementary Figure 3, and Supplementary Tables 2A,B). These data suggest that the AGO-loading efficiency of these miRNAs can be different in dark-grown and de-etiolated seedlings. Different AGO loading can compensate for the significantly different accumulation of miRNAs in the inputs and stabilize the active miRNA levels during de-etiolation (miR842, miR5654-3p, miR1886.1, and miR779.2) or could lead to significantly different active miRNA amounts even though that the input levels were similar (miR824-5p, miR156f-3p, miR5651, miR396b-5p, miR168a-5p, and miR157b-5p). Six miRNAs were present in significantly different amounts in both the inputs and RISC-loaded samples (miR773a, miR408-5p, miR319a, miR167d, miR399c-3p, and miR163) (Figure 4A and Supplementary Table 2C). In five of them (miR773a, miR408-5p, miR319a, miR167d, and miR399c-3p), the differences were similar, thus these miRNAs are loaded onto RISC with similar efficiency in dark-grown and de-etiolated seedlings. By contrast, miR163 was loaded onto RISC much more effectively in light; miR163 was 20-fold more abundant in the light input relative to the dark input, while the difference was increased to 109.6-fold at RISC-loaded samples (Supplementary Table 2C). Similar results were obtained when the RISC-loading efficiency was measured by comparing RISC-loaded miRNA values/input miRNA values in dark and light samples (Supplementary Figure 3).

Taken together, RISC-loading efficiencies are similar for most miRNAs in both dark-grown and de-etiolate seedlings, moderately changed for certain miRNAs, and considerably increased for miR163.

### Argonaute 1 and MicroRNAs Are Similarly Partitioned in Dark-Grown and De-Etiolated Plants

Previous comparative miRNAome, transcriptome, and degradome studies indicated that miRNA cleavage activity is increased in de-etiolated plants (Lin et al., 2017). As our results show that both total and RISC-loaded miRNAomes (except miR163) are similar in dark-grown and de-etiolated seedlings, it is unlikely that more efficient RISC-loading leads to more intense cleavage in light. Light stimulated cleavage could be explained if the nucleocytoplasmic shuttling protein AGO1 and the AGO1-loaded miRNAs are more efficiently exported from the nucleus in de-etiolated plants. To test this assumption, cytoplasmic and nuclear fractions were separated from crude extracts of dark-grown and de-etiolated plants, and then AGO1 protein and miR168 and miR159 levels were studied in the fractions (miR163 was not analyzed as it is not detectable in the dark samples). The fractionation was efficient, and the nuclear and the cytoplasmic control proteins (H3 and actin) were only present in the corresponding fractions (Figure 4B). Relevantly, we found that nuclear-cytoplasmic distribution of AGO1 was not significantly different in the dark-grown and de-etiolated plants (although it was slightly overrepresented in the cytoplasm in light). Moreover, miR168 and miR159 were also comparably partitioned (Figure 4B). These data do not support the model that different distribution of AGO1 and AGO1-loaded miRNAs would be responsible for the proposed light-enhanced miRNA activity.

## Discussion

Previous studies reported conflicting results about the role of miRNAs in the regulation of photomorphogenesis. While findings that miRNA biogenesis factors and certain miRNAs are required for normal photomorphogenesis and that miRNA-guided RISC cleavage activity is increased during de-etiolation indicate that miRNA regulation plays an important role in the control of photomorphogenesis, and the results that the total miRNAome is only slightly changed during de-etiolation suggest that miRNAs do not play a critical regulatory role (Choi et al., 2020; Li et al., 2021). These studies compared total miRNAomes during de-etiolation supposing that it represents the functional miRNAome. However, RISC-loading is regulated in *Arabidopsis*, thus RISC-loaded miRNAome could be different from total miRNAome (Dalmadi et al., 2019). Therefore, we compared the changes in the total miRNAome and the RISC-loaded miRNAome during photomorphogenesis. RISC-loaded miRNAome was selected by isolating miRNAs that co-fractionated with AGO1 (Figure 1). The previous study has shown (and our study supports) that miRNAs are present in miRNA duplex form in the low mol. weight fractions or in the AGO1 containing high mol. weight fractions (Dalmadi et al., 2019). This suggests that most miRNAs are loaded onto AGO1 and/or that, all miRNA harboring RISCs accumulate in the high mol. weight fractions even if they contain different AGOs (except AGO4 that contains siRNAs and accumulates in the medium weight fractions). It is proposed (Dalmadi et al., 2019) that AGO1-containing fractions represent the biologically active miRNA pool, thus sequencing of these fractions is an efficient method to characterize the active, RISC-loaded miRNAome (see at the end of the discussion the limitation of the method).

In line with other studies (Choi et al., 2020; Li et al., 2021), we found that total miRNAome altered modestly during de-etiolation, miRNA163 was dramatically upregulated, while few other miRNAs show only moderate changes (Figure 2). These subtle expression differences are not easily reproducible, for instance, a recent study reported that only miR163 showed significantly different expression during photomorphogenesis (Li et al., 2021), while we and others found that many miRNAs expressed weakly but significantly different between dark-grown and de-etiolated plants (Choi et al., 2020). The differences in the experimental setup and growing conditions (for instance, we used 8 days dark-grown seedlings while another similar study used 5 days old dark-grown plants) might be responsible for the inconsistencies (Choi et al., 2020). Interestingly, while seedling de-etiolation, which mimics reaching the soil surface, leads to moderate total miRNAome changes, 3 days of dark treatment of long day adapted plant, which simulates reburial, results in a dramatic reduction in miRNA expression (Achkar et al., 2018). While de-etiolation leads to the accumulation of microprocessor components in mainly inhibited complexes, dark shift results in the rapid reduction of HYL1 protein levels (Achkar et al., 2018; Park et al., 2021; Jung et al., 2022).

If RISC-loading is regulated during de-etiolation, the RISC-loaded miRNAomes of dark-grown and de-etiolated plants should be more different than the total miRNAomes. However, we have found that the RISC-loaded miRNAomes of dark-grown and de-etiolated seedlings are similar (Figure 3 and Supplementary Table 2A), suggesting that RISC-loading efficiency is changed only slightly during photomorphogenesis. miR163 was the exception, it was RISC-loaded much more efficiently in the de-etiolated plant. RISC-loading altered the representation of 10 miRNAs slightly but significantly (Figure 4A), four were differentially present in the total miRNAomes but not in the RISC-loaded miRNAome, while six miRNAs were significantly differentially represented only in the RISC-loaded miRNAomes. These data suggest that during de-etiolation, regulated RISC-loading does not fundamentally control the formation of biologically active miRNAome (except miR163, see below) but could contribute to the fine-tuning of miRNA expression. If the export of RISC-loaded miRNAs from the nucleus is more effective in de-etiolated plants, the increased target cleavage activity in the de-etiolated seedlings could be explained even if the RISC-loaded miRNAomes of dark-grown and de-etiolated plants are similar. However, we found that AGO1 and the two studied miRNAs (miR159 and miR168) were present in a comparable amount in the nuclear and cytoplasmic fractions in both the dark-grown and de-etiolated samples (Figure 4B). These data suggest that AGO-loaded miRNA export operates similarly during de-etiolation. High-throughput sequencing of nuclear and cytoplasmic fractions during de-etiolation could confirm this conclusion. Taken together, our results indicate that neither regulated RISC-loading nor differences in nuclear export of RISC cannot explain the apparent conflict between the modest miRNAome changes during de-etiolation and the relevant role of miRNA biogenesis factors in normal photomorphogenesis (Sun et al., 2018). Further studies are required to clarify whether miRNA-based regulation is not important for photomorphogenesis or several subtle changes in miRNA expression, loading, and export are physiologically relevant and required for the fine-tuning of gene expression during de-etiolation.

It might be surprising as regulated RISC-loading plays a critical role in forming leaf and flower-specific active miRNAomes (Dalmadi et al., 2019). However, miRNA regulation likely plays a more important role in the control of leaf and flower development than in de-etiolation, thus their biologically active miRNAomes should be more different. Indeed, the total miRNAome was already far different between leaves and flowers and it was further modified by organ-specific regulated RISC-loading (Dalmadi et al., 2019).

MiR163 was an exception, as RISC-loading of miR163 is much more efficient in de-etiolated than in the dark-grown plants. miR163 is a highly specific miRNA; it is an evolutionary young 24 nt long miRNA, whose expression is dramatically enhanced during de-etiolation (Chung et al., 2016; Choi et al., 2020; Li et al., 2021). This increased expression is required for normal photomorphogenesis, and miR163-mediated silencing of PXMT1 methyltransferase promotes primary root growth (Chung et al., 2016; Li et al., 2021). miR163 overexpression during de-etiolation is regulated at different steps including transcriptional activation (Choi et al., 2020) and more efficient RISC-loading (this study). Pri-miR163 is increased to a much higher level (>100-fold) than any other pri-miRNA during de-etiolation (Choi et al., 2020). Its transcription is dramatically activated because the miR163 promoter contains strong binding sites for LONG HYPOCOTYL 5 (HY5) light-activated transcription factor (Li et al., 2021). We found that it was overrepresented >5-fold in the RISC-loaded miRNAome relative to the total miRNAome in the de-etiolated sample (but not in the dark-grown plant) showing that the RISC-loading efficiency of miR163 is much higher in light (Figure 3, Supplementary Figure 3, and Supplementary Table 2C). We propose that a high active miR163 level is required for normal photomorphogenesis and that the enhanced RISC-loading of miRNA163 plays a critical role to reach this high miR163 concentration. RISC-loading efficiency depends on (i) how effectively the miRNA incorporates into RISC and on (ii) the stability of the miRNA in the RISC complex. It was suggested that in light, the degradation of many miRNAs is accelerated, while the stability of miR163 is not altered (Choi et al., 2020). If RISC-loaded miRNA163 is much more stable in light than the other RISC-loaded miRNAs, it could explain the overrepresentation of miRNA163 in the de-etiolated miRNAome. A more likely (but not mutually exclusive) explanation is that RISC incorporation of miR163 is increased in de-etiolated plants. RISC-loading efficiency has been studied only for miR168. It was shown that the secondary structure of precursor RNA defines the efficiency of RISC incorporation (Dalmadi et al., 2021). A light-dependent factor may modify the splicing or secondary structure formation of miR163 precursor, thereby facilitating the incorporation of miRNA163 into RISC in de-etiolated plants. Indeed, the miR163 precursor is unusually long and its splicing is required for efficient miRNA generation (Bielewicz et al., 2013).

### Characterization of Active miRNAome

This study is based on the assumption that miRNAs that co-fractionate with AGO1 are present in active RISC complexes. However, a fraction of them may be present in (same-sized) other complexes (Dalmadi et al., 2019). Moreover, as the crude extracts contain both cytoplasmic and nuclear components, part of miRNAs in the high mol. weight gel-filtrated fractions could be present in inactive, nuclear RISC complexes. Although our assays suggest that the export of RISC-loaded miRNA act with similar efficiency in dark-grown and de-etiolated plants, it might be different under other experimental conditions. Thus, separation of cytoplasmic fraction followed by gel-filtration might be a more accurate approach to identify active miRNAome. An alternative way to identify active miRNAome is AGO1 IP and sequencing of co-immunoprecipitated sRNAs (AGO1 RIP-seq) (Trolet et al., 2019). Both methods have advantages and disadvantages, AGO1 RIP-seq is selective but fails to detect miRNAs that are present in other AGOs, while gel-filtration could contain all AGO-bound miRNAs but it is less selective (Dalmadi et al., 2019). As it is not known, which AGOs play important role in de-etiolation, we choose the more general gel-filtration method to select the active miRNAome.

## Data Availability Statement

The datasets presented in this study can be found in online repositories. The names of the repository/repositories and accession number(s) can be found below: https://www.ncbi.nlm.nih.gov/, PRJNA795329.

## Author Contributions

LL and FN designed the study. LL conducted the experiments. GG and LL analyzed the sequencing data. LL, DS, and FN wrote the manuscript. All authors contributed to the article and approved the submitted version.

## Conflict of Interest

The authors declare that the research was conducted in the absence of any commercial or financial relationships that could be construed as a potential conflict of interest.

## Publisher’s Note

All claims expressed in this article are solely those of the authors and do not necessarily represent those of their affiliated organizations, or those of the publisher, the editors and the reviewers. Any product that may be evaluated in this article, or claim that may be made by its manufacturer, is not guaranteed or endorsed by the publisher.

## Supplementary Material

Supplementary Figure 1 MiR163, miR159b, and miR168 are associated with AGO1. **(A,B)** Shows the remaining two replicas of Figure 1C, therefore the description is as in Figure 1C. Statistical analysis of the three replicas is provided in Figure 1C.

Supplementary Figure 2 Principal component analysis (PCA) of total miRNAome and RISC-loaded miRNAome of dark-grown and de-etiolated *Arabidopsis* seedling. PCA shows that the four miRNAomes are well separated. High throughput miRNA sequencing was conducted from crude extracts of dark-grown and de-etiolated seedlings (D. input and L. input, respectively) and from AGO1-containing fractions of gel-filtrated D and L samples (D. RISC-loaded and L. RISC-loaded).

Supplementary Figure 3 Regulated RISC-loading moderately controls the active miRNAome. **(A,B)** To illustrate the role of regulated RISC-loading in the formation of active, AGO-loaded miRNAome, the RISC-loaded miRNA mean value/Input miRNA mean values of de-etiolated (Light) and dark-grown (Dark) seedlings were compared and the results are presented in **(A)** heat-map and **(B)** relative miRNA distribution forms.

Supplementary Figure 4 Distribution of AGO1 and miRNAs in the crude extracts of *A. thaliana* seedlings. Nuclear and cytoplasmic fractions were separated from crude extracts of dark-grown (Dark) and de-etiolated (Light) seedlings. RNA and protein samples were isolated from the cytoplasmic fraction (C), the third wash (W#3), and the isolated nuclei (N) and then subjected to Western and Northern analysis. AGO1, nuclear control histone H3, and cytoplasmic control actin proteins were detected. miR168 and miR159b were detected by Northern hybridization. Statistical analysis of the three replicas is provided in Figure 4B.

Supplementary Table 1 **(A)** Shows miRNAs identified by RNA-seq in all samples. We presented four replicate samples from each condition (RISC light sample replicates 1–4, RISC dark sample replicates 1–4, Input light sample replicates 1–4, and Input dark sample replicates 1–4). **(B)** Contains the list of miRNAs that were applied for analyses in our study. **(C)** Shows miRNA that was excluded from the analyses including miRNA*s. **(D)** Contains duplicated miRNAs. **(E)** displays those miRNAs that were excluded based on low abundance.

Supplementary Table 2 miRNAs, whose expression is affected by de-etiolation. **(A,B)** List of miRNAs that are differentially present in the **(A)** inputs or **(B)** in the RISC-loaded samples of dark-grown and de-etiolated seedlings (dark and light, respectively). miRNAs with at least ± 2 fold difference are highlighted in red. FC, fold change. **(C)** miR163 is the only miRNA, which is overrepresented in both Input and RISC-loaded light samples **(A,B)**, in which RISC-loading is different in dark and light (FC value of **B**/FC value of **A**).
