# Supplementary figures and images for: In Arabidopsis thaliana, RNA-Induced Silencing Complex-Loading of MicroRNAs Plays a Minor Regulatory Role During Photomorphogenesis Except for miR163
Source: Front Plant Sci. 2022 Jul 13;13:854869. doi: 10.3389/fpls.2022.854869 (PMC9326452; doi:10.3389/fpls.2022.854869)

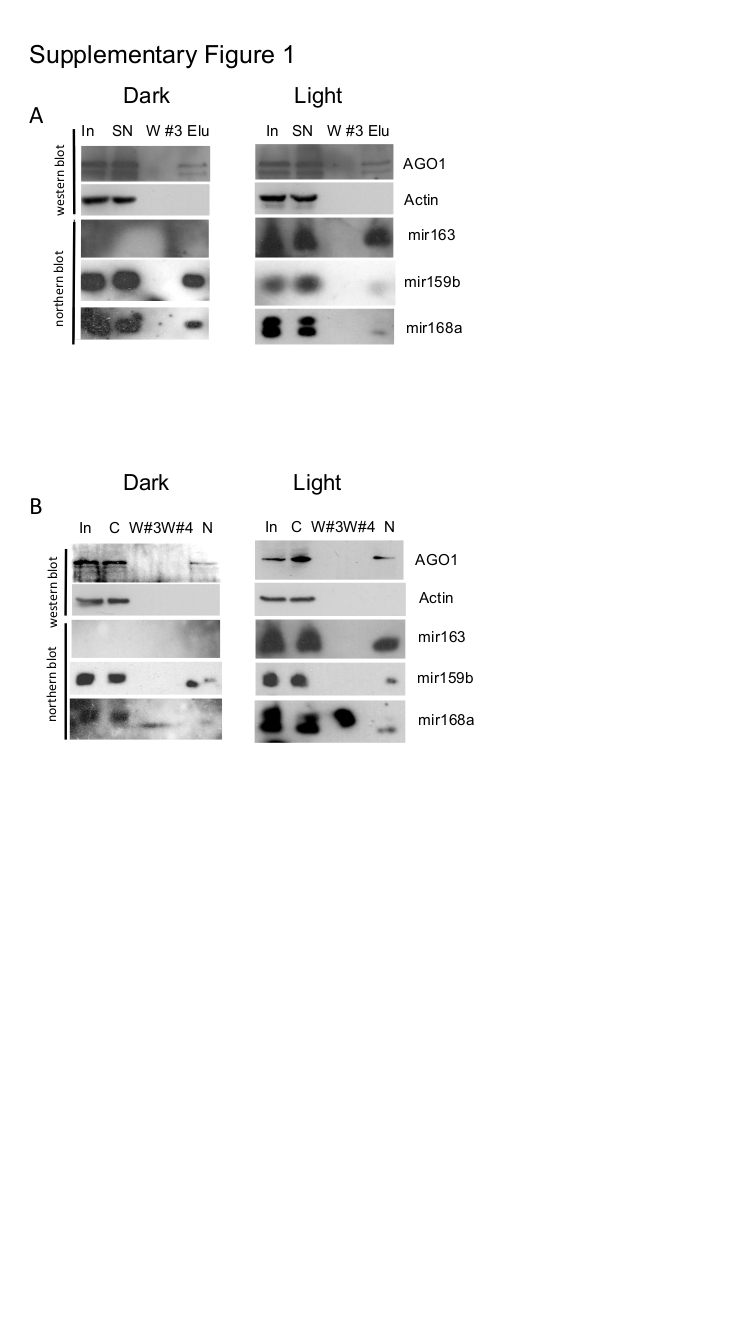

Supplement: Supplementary Figure 1 — MiR163, miR159b, and miR168 are associated with AGO1. (A,B) Shows the remaining two replicas of Figure 1C, therefore the description is as in Figure 1C. Statistical analysis of the three replicas is provided in Figure 1C. [file Image_1.jpeg]

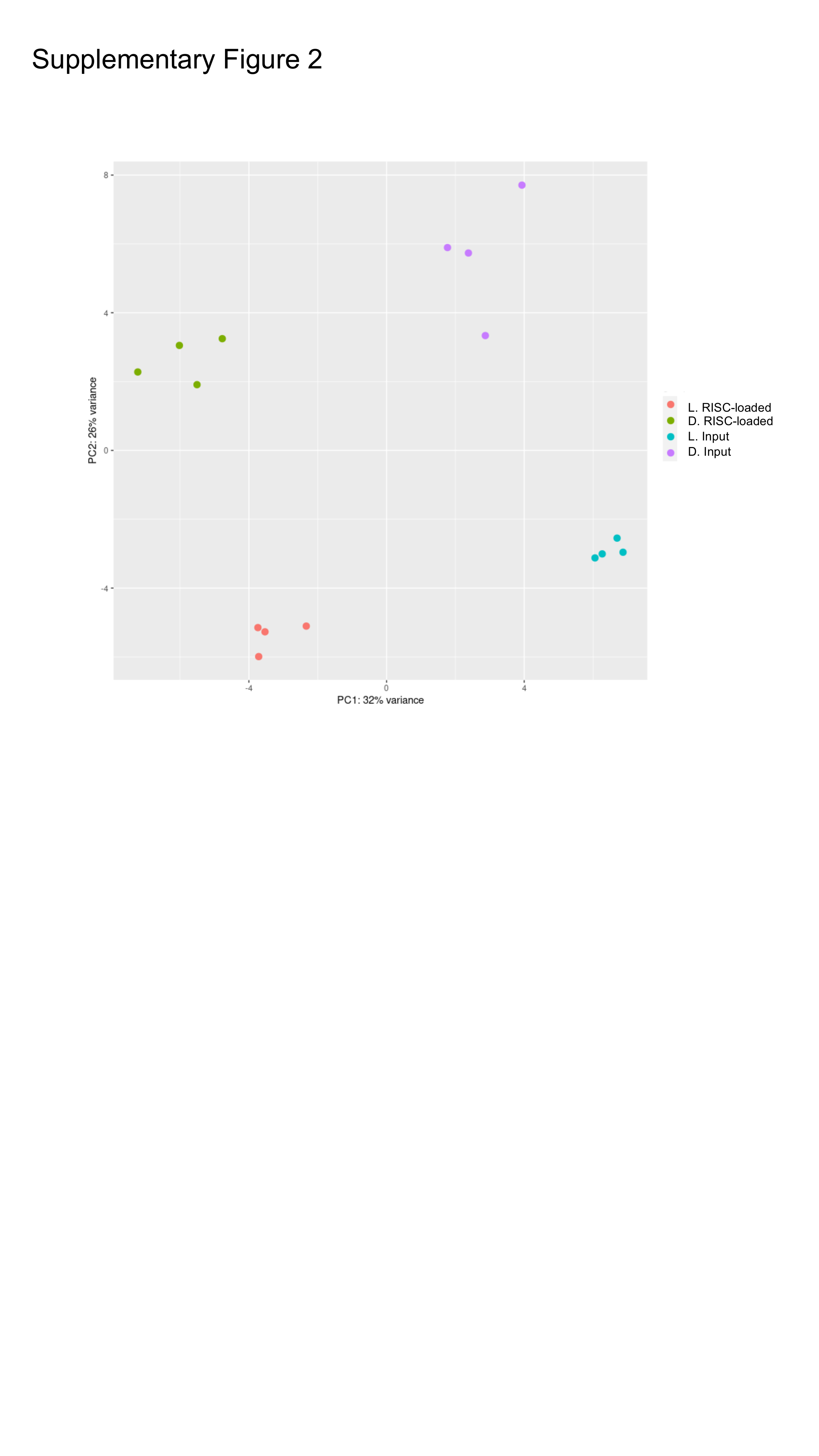

Supplement: Supplementary Figure 2 — Principal component analysis (PCA) of total miRNAome and RISC-loaded miRNAome of dark-grown and de-etiolated Arabidopsis seedling. PCA shows that the four miRNAomes are well separated. High throughput miRNA sequencing was conducted from crude extracts of dark-grown and de-etiolated seedlings (D. input and L. input, respectively) and from AGO1-containing fractions of gel-filtrated D and L samples (D. RISC-loaded and L. RISC-loaded). [file Image_2.jpeg]

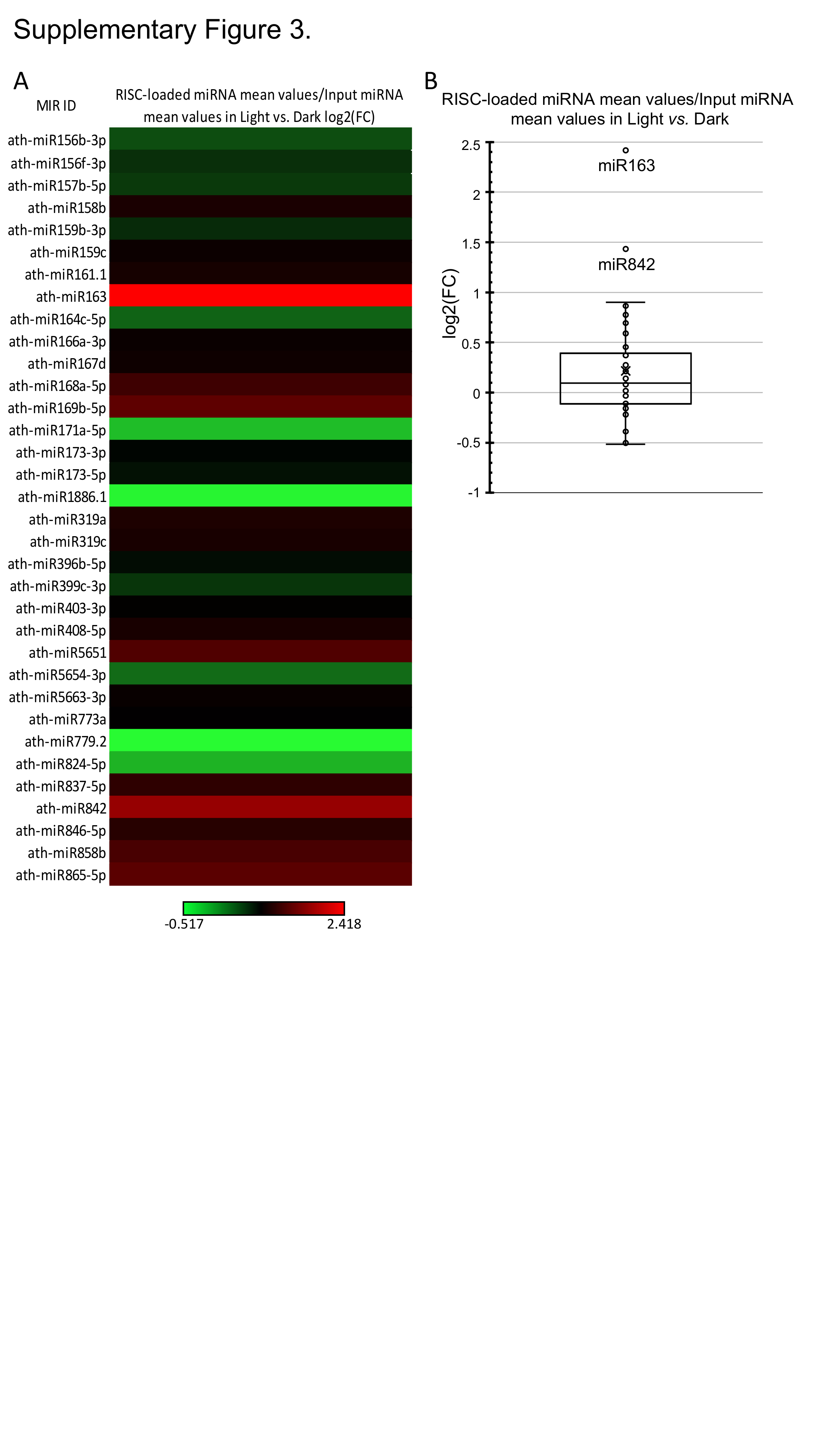

Supplement: Supplementary Figure 3 — Regulated RISC-loading moderately controls the active miRNAome. (A,B) To illustrate the role of regulated RISC-loading in the formation of active, AGO-loaded miRNAome, the RISC-loaded miRNA mean value/Input miRNA mean values of de-etiolated (Light) and dark-grown (Dark) seedlings were compared and the results are presented in (A) heat-map and (B) relative miRNA distribution forms. [file Image_3.jpeg]

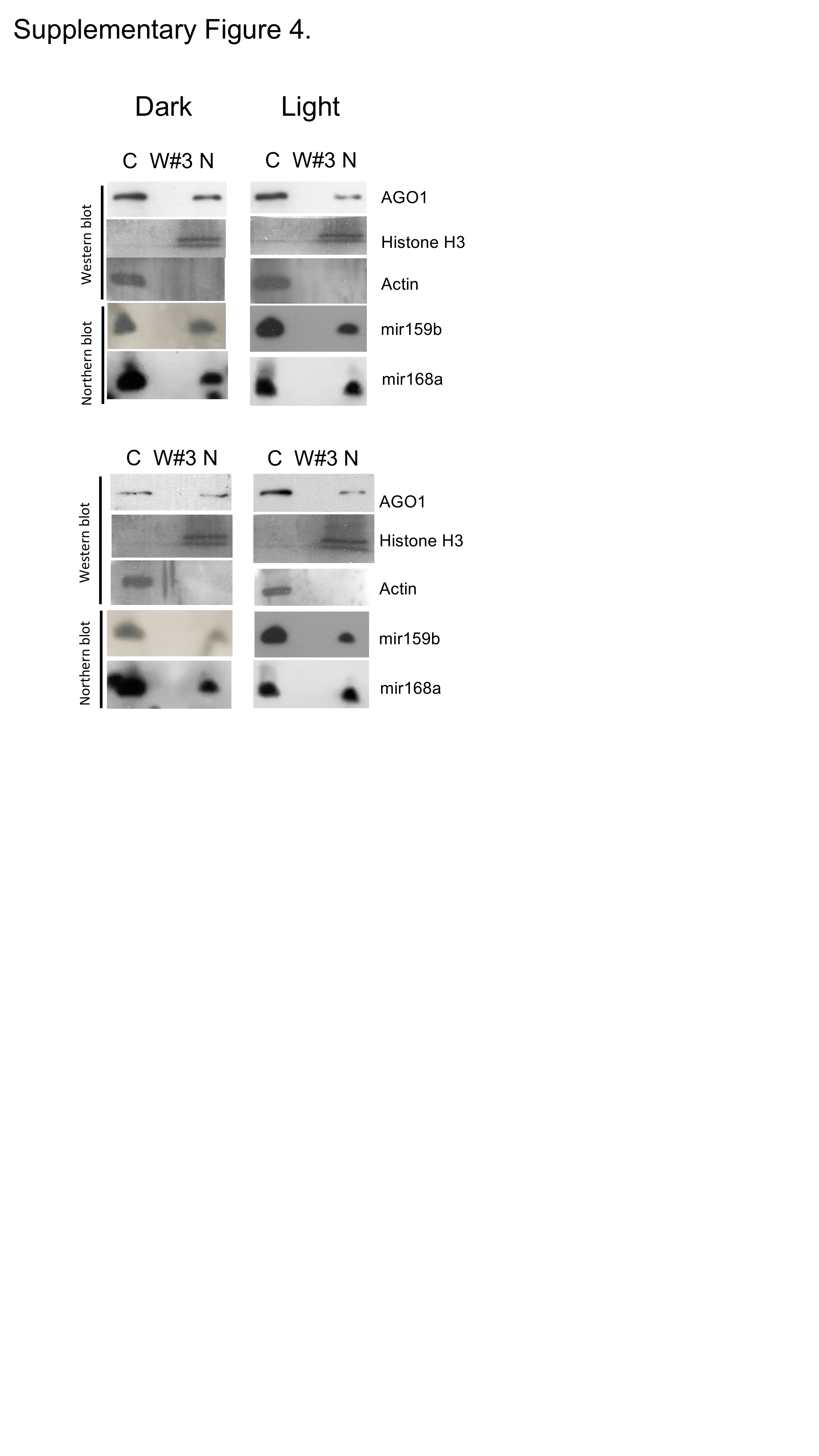

Supplement: Supplementary Figure 4 — Distribution of AGO1 and miRNAs in the crude extracts of A. thaliana seedlings. Nuclear and cytoplasmic fractions were separated from crude extracts of dark-grown (Dark) and de-etiolated (Light) seedlings. RNA and protein samples were isolated from the cytoplasmic fraction (C), the third wash (W#3), and the isolated nuclei (N) and then subjected to Western and Northern analysis. AGO1, nuclear control histone H3, and cytoplasmic control actin proteins were detected. miR168 and miR159b were detected by Northern hybridization. Statistical analysis of the three replicas is provided in Figure 4B. [file Image_4.jpeg]
