# Supplementary material for: In Arabidopsis thaliana, RNA-Induced Silencing Complex-Loading of MicroRNAs Plays a Minor Regulatory Role During Photomorphogenesis Except for miR163
Source: Front Plant Sci. 2022 Jul 13;13:854869. doi: 10.3389/fpls.2022.854869 (PMC9326452; doi:10.3389/fpls.2022.854869)
Supplement: Supplementary Table 2 — miRNAs, whose expression is affected by de-etiolation. (A,B) List of miRNAs that are differentially present in the (A) inputs or (B) in the RISC-loaded samples of dark-grown and de-etiolated seedlings (dark and light, respectively). miRNAs with at least ± 2 fold difference are highlighted in red. FC, fold change. (C) miR163 is the only miRNA, which is overrepresented in both Input and RISC-loaded light samples (A,B), in which RISC-loading is different in dark and light (FC value of B/FC value of A). [file Table_2.pdf]

Supplementary Table 2

A

Input miRNAs with different amounts in Light and Dark			
MIR ID	FC	log2(FC)	p-value
ath-miR163¥	20.523	4.359	0.00017
ath-miR167d¥	0.631	-0.663	0.03
ath-miR1886.1¥	1.636	0.71	0.0026
ath-miR319a¥	0.498	-1.006	0.0128
ath-miR399c-3p¥	1.744	0.803	0.0049
ath-miR408-5p¥	0.378	-1.402	0.0042
ath-miR5654-3p¥	1.443	0.53	0.0048
ath-miR773a¥	0.541	-0.885	0.0118
ath-miR779.2¥	1.528	0.612	0.0321
ath-miR842¥	0.47	-1.088	0.000073

B

miRNAs with different RISC-loading in Light and Dark			
MIR ID	FC	log2(FC)	p-value
ath-miR156f-3p¥	0.442	-1.179	0.009
ath-miR157b-5p¥	1.328	0.409	0.01
ath-miR163¥	109.668	6.777	6.503E-07
ath-miR167d¥	0.696	-0.522	0.041
ath-miR168a-5p¥	1.418	0.504	0.008
ath-miR319a¥	0.603	-0.731	0.003
ath-miR396b-5p¥	1.213	0.279	0.013
ath-miR399c-3p¥	1.619	0.695	0.016
ath-miR408-5p¥	0.444	-1.17	0.003
ath-miR5651¥	2.206	1.142	0.009
ath-miR773a¥	0.549	-0.866	0.004
ath-miR824-5p¥	0.78	-0.359	0.027

C

Ratio of FC of RISC-loaded/Input miRNAs	
MIR ID	values
ath-miR163¥	5.33
ath-miR399c-3p¥	0.91
ath-miR167d¥	1.10
ath-miR319a¥	1.21
ath-miR773a¥	1.01
ath-miR408-5p¥	1.17
